# Modeling pathological brain rhythms: constructing a neural mass model from single cell dynamics

**DOI:** 10.1186/1471-2202-14-S1-P383

**Published:** 2013-07-08

**Authors:** Bas-Jan Zandt, Sid Visser, Michel JAM van Putten, Bennie ten Haken

**Affiliations:** 1 Neuroimaging, University of Twente, Enschede, 7500AE, the Netherlands; 2 Applied Analysis and Mathematical Physics, University of Twente, Enschede, 7500AE, the Netherlands; 3 Clinical Neurophysiology, University of Twente, Enschede, 7500AE, the Netherlands; 4 Department of Clinical Neurophysiology, Medisch Spectrum Twente, Enschede, the Netherlands

## 

Neural mass models (NMM) describe neural activity on a macroscopic scale, which can be compared to the electroencephalogram (EEG). This allows a better understanding of the processes responsible for various EEG patterns, including pathological rhythms as diffuse slowing or burst-suppression [1].

Using available models which contain explicit expressions for the synaptic response and number of synapses [2], pathological conditions that modulate synaptic function, such as anesthetics [3] and hypoxia, can be included easily. However, it is less obvious how to incorporate conditions which alter the excitability of neurons, such as hyperkalemia or channel blockers.

Here, we present a method for constructing a neural mass model by using the relation between synaptic input of a single cell model and its firing rate. This allows an easy implementation for pathological conditions.

We describe the average firing rate of a single population of neurons receiving one type of synaptic input, but this can readily be extended to multiple populations. A set of differential equations describes, traditionally, the average synaptic conductance [2]. Assuming Poisson statistics for the input, we can derive another equation, which describes the time evolution of the standard deviation of the synaptic conductance across the population. The average and standard deviation of the conductance then determine the distribution and the corresponding average of the firing rates in the population.

**Figure 1:**
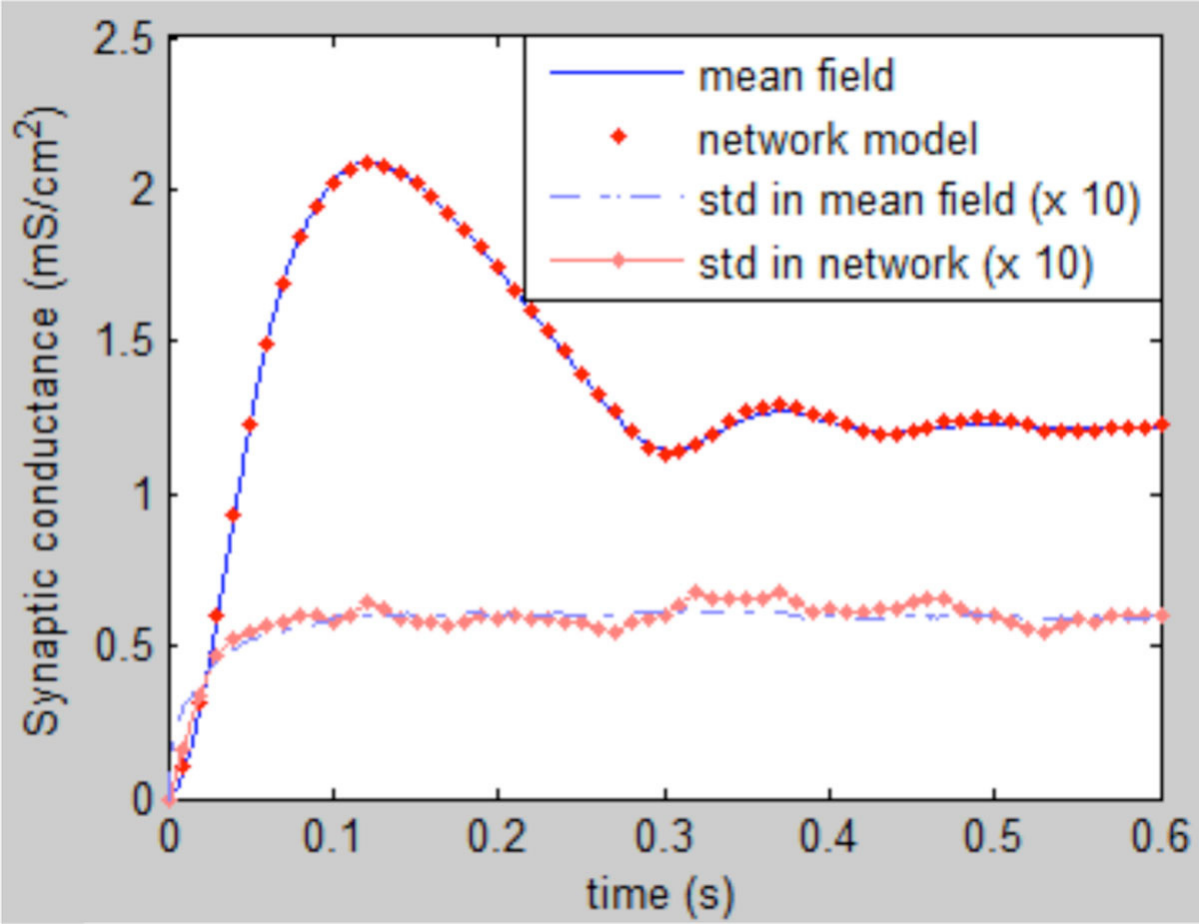
Comparison of step response of the derived NMM and a detailed network model

As initial verification, the constructed mean field model is numerically compared to a network of single cells. From the single cell model we determine the dependence of the firing rate on (constant) synaptic conductance numerically. Furthermore, we show that, for fluctuating inputs, the firing rate is well approximated by the instantaneous synaptic conductance. 120 Hodgkin-Huxley type cells were connected all-to-all with inhibitory synapses: a simple configuration which results in intrinsic oscillations. Each cell receives inhibitory external input as well, consisting of Poisson trains. We find a close agreement between the constructed neural mass model and the network simulation (Figure 1).

The proposed method can easily be extended to model heterogeneous populations, multiple types of synapses, spatial structures, propagation delays, and bursting dynamics [4]. Any pathophysiology can readily be incorporated by adapting the single cell model. This allows for testing hypotheses on processes underlying abnormal EEGs.
